# Epigenetic Variability Confounds Transcriptome but Not Proteome Profiling for Coexpression-based Gene Function Prediction*[Fn FN2]

**DOI:** 10.1074/mcp.RA118.000935

**Published:** 2018-07-24

**Authors:** Piotr Grabowski, Georg Kustatscher, Juri Rappsilber

**Affiliations:** From the ‡Bioanalytics, Institute of Biotechnology, Technische Universität Berlin, 13355 Berlin, Germany;; §Wellcome Centre for Cell Biology, University of Edinburgh, Edinburgh EH9 3BF, UK

**Keywords:** Systems biology, Gene Expression, Epigenetics, Chromatin function or biology, Computational Biology, gene expression variability, gene function prediction, genome organisation, proteomics, transcriptomics

## Abstract

Genes are often coexpressed with their genomic neighbors, even if these are functionally unrelated. For small expression changes driven by genetic variation within the same cell type, non-functional mRNA coexpression is not propagated to the protein level. However, it is unclear if protein levels are also buffered against any non-functional mRNA coexpression accompanying large, regulated changes in the gene expression program, such as those occurring during cell differentiation. Here, we address this question by analyzing mRNA and protein expression changes for housekeeping genes across 20 mouse tissues. We find that a large proportion of mRNA coexpression is indeed non-functional and does not lead to coexpressed proteins. Chromosomal proximity of genes explains a proportion of this nonfunctional mRNA coexpression. However, the main driver of non-functional mRNA coexpression across mouse tissues is epigenetic similarity. Both factors together provide an explanation for why monitoring protein coexpression outperforms mRNA coexpression data in gene function prediction. Furthermore, this suggests that housekeeping genes translocating during evolution within genomic subcompartments might maintain their broad expression pattern.

Genes are not arranged randomly but tend to be clustered in the genome into coexpressed domains (1). Such clustering can be a regulatory strategy of both prokaryotic and eukaryotic genomes. Interestingly, this does not mean that genes that are coexpressed are necessarily also linked functionally. There exist gene clusters that tend to be coexpressed, yet lack evident cofunctionality (1, 2). This is especially visible for bidirectional gene pairs which are coexpressed because of shared regulatory context, but commonly seem to lack a functional relationship (3). This has an impact on gene coexpression studies which infer functional associations between genes based on similar gene activity. Coexpression of spatially close genes can be driven by stochastic transcriptional bursting (4) or transcriptional interference between neighboring genes (5). The existence of coexpressed gene clusters that lack a functional connection is intriguing given that nonspecific gene expression should have a negative impact on cell fitness. Interestingly, Hurst and colleagues have shown that clustered genes mutually reinforce their active state and are less likely to be accidentally silenced, for example by stochastic fluctuations of chromatin states (6). Therefore, clustered genes show lower expression noise, a benefit that may offset the negative impact of their coincidental coexpression. In agreement with this, we have recently demonstrated that coexpression of proximal genes, both in terms of sequence and 3D genomic proximity, is pervasive in the human genome. Importantly, however, coexpression of spatially close, functionally unrelated genes is restricted to their mRNA abundances and is not propagated to the protein level (7). This protein-level buffering of non-functional mRNA coexpression supports the idea that reduction of expression noise is a key driver of the evolution of genome organization. Consequently, function prediction is based better on protein coexpression than mRNA coexpression data (8, 9).

Our previous analysis was based on a panel of human lymphoblastoid cell lines (LCLs)^1^ for which the expression changes had a prominent noise component owing to the little variability between the cell lines. A related analysis of human cancer panels also found mRNA—but not protein—coexpression to reflect chromosomal gene colocalization (8). However, it remains to be seen if a similar uncoupling of transcriptome and proteome exists also for strong, regulated and biologically important expression changes. For example, different cell types have different metabolic needs, morphology, organelle numbers and sizes. Even for ubiquitously expressed housekeeping genes, this can amount to large quantitative differences in expression levels. Here, we investigate the impact of genome organization and epigenetic states on mRNA and protein coexpression across different mouse tissues by integrating multiple published omics data sets. We show that the observations made on cell lines regarding factors governing mRNA and protein coexpression also hold in tissues, with changes in the relative weights of the contributions from genome position *versus* epigenetic state. We point at possible biases in expression profiling for functional genomics that researchers should consider.

## EXPERIMENTAL PROCEDURES

### 

#### 

##### Mouse Tissue mRNA and Protein Expression Data Set Assembly

SILAC mouse tissue proteomes were downloaded from (10), normalized SILAC H/L ratios for each tissue extracted and log2-transformed. SILAC kidney values were obtained by averaging expression values for kidney cortex and medulla.

Transcriptomics profiling data of tissues were obtained from (11–15) (links in supplemental Table S1). Data downloaded from ENCODE were in Gencode M4-aligned bam format with the only exception of the skeletal muscle data which were downloaded in fastq format and aligned using TopHat v2.0.9 and Gencode M4 annotation. The TopHat settings were set to default apart from using “bowtie1” parameter and library type set to “fr-secondstrand.” The bam files were subsequently processed using Cufflinks 2.2.1 with default settings to obtain gene expression (fragments per kilobase of exon model per million mapped reads, FPKM) values. The three tissues downloaded from GEO were in normalized FPKM or RPKM format. All the mRNA expression data were transformed into a common transcripts per million (TPM) unit. To make the RNAseq data set comparable with the proteomics data, each mRNA expression value was divided by a median expression value for a given gene in all 20 tissues (analogously to the Super-SILAC approach (16) used in the proteomics data set). Finally, the normalized TPM ratios were log2-transformed.

The resulting mRNA and protein expression data set contains 3391 genes with expression values in at least 8 tissues on both mRNA and protein levels. The proteomics data and mRNA data contain 15.5% and 6.7% missing values, respectively.

The processed data set is available as supplemental File S1.

##### Epigenetics Data Processing

ChIPseq data for 9 mouse tissues (marks: H3K27ac, H3K27me3, H3K36me3, H3K4me1, H3K4me3, H3K79me2) were obtained from ENCODE in bigWig format (fold change *versus* control). The data for H3K9ac was available only for two tissues. To extract mean ChIPseq signal per gene body for all tissues, a UCSC bigWigAverageOverBed command line tool was used in conjunction with a custom-made bed file based on Gencode M4 mouse gene annotation. The processed ChIPseq data set is available as supplemental File S2.

##### Gene Expression Correlation Analysis

To obtain the between-gene correlation values the data were centered at 0 for each experiment and a Pearson correlation coefficient was calculated using R function “corr.test” from the psych package with the “use” parameter set to “pairwise.complete.obs.” For improved statistical power, correlations were calculated only for genes which had data in at least 8 overlapping tissues (both on protein and mRNA levels). Gene pairs were considered correlated if their PCC value was > 0.5. For subsequent analyses, only correlations with Benjamini-Hochberg adjusted *p* values < 0.05 were considered.

##### Genomic Positions of Genes and Intergenic Distances

Mouse gene positions on mm10 genome were obtained from Ensembl Biomart (17, 18) (state on 29.06.2017). For gene distance calculation, first base pair of each gene's outermost transcription start site (TSS) was used and distances between those positions calculated for each gene pair.

##### Statistical Significance Analysis of Close-by and Other Coregulated Genes

Two Pearson Chi-squared tests were performed on two 2 × 2 contingency tables (for mRNA and protein levels). The first contingency table (mRNA-level) divided gene pairs by two variables. The first variable considered genomic distances between the gene pairs (close-by/other) and the second variable divided the gene pairs according to their mRNA coexpression (gene pairs with mRNA Pearson correlation coefficient > 0.5 and BH-adjusted *p* value < 0.05 were considered correlated and all other pairs were considered uncorrelated). Similarly, for the protein-level analysis, the first variable was genomic proximity. In the second variable, pairs were correlated if they both had mRNA and protein PCC > 0.5 and the BH-adjusted *p* value < 0.05.

##### Analysis of Post-transcriptional Mechanisms

The miRNA/gene mapping data for mouse brain were obtained from (19). The CDS lengths of coexpressed genes were obtained from Biomart using Ensembl Genes 92 database and the GRCm38.p6 data set. The genes were considered to have similar CDS length if the ratio of the length of the longer CDS to the shorter CDS was below 1.5. The liver time-series ribosome profiling data was obtained from (20). Ribosome profiling matrices were scaled using the accompanying mRNA expression data and the resulting ratios were log2-transformed. Finally, Pearson correlation coefficients between genes were calculated using R function “corr.test” from the “psych” package (21). Gene pairs with Pearson correlation coefficient > 0.5 and the Holm-adjusted *p* value < 0.001 were considered as correlated. Protein translation rates were obtained from (22). For each gene pair, a ratio of their translation rates was calculated, log2-transformed and the absolute values taken. Gene pairs were considered to have similar translation rates if this absolute log2 ratio was lower or equal to 1. The protein degradation profiles were obtained from (23) and gene pairs coding at least one nonexponentially degraded protein were counted.

##### K-means Clustering of mRNA and Protein Expression Data

The Pearson correlation coefficients for all gene pairs were used to cluster the mRNA and protein data separately. An R clustering function “kmeans” was used for this purpose. The first k value that explained 50% of the variance in the data was selected. The percentage of variance explained was defined as the ratio of the between sum of squares to the total sum of squares for every given k. The parameter “nstart” was set to 3 and “max.iter” set to 20.

##### Subcellular Localization Enrichment

Subcellular localization annotation was obtained from Uniprot (24). Proteins localized to more than one subcellular compartment were removed. Endoplasmic reticulum was joined with Golgi as “ER/Golgi” to balance the group sizes. Only “nucleus,” “mitochondrion,” and joined “ER/Golgi” groups were considered for subcellular localization enrichments. The expected value for each cluster was defined as the percentage of proteins with the given subcellular localization annotation in the data. The observed value was calculated as a percentage of those proteins in the given cluster. Finally, log2 observed/expected values were calculated for each of the cluster and subcellular localization.

##### GO Enrichment Analysis

Gene Ontology enrichments were performed using DAVID online service (25). All Uniprot Accession numbers belonging to each of the clusters were used as a query and the whole mouse genome used as background for statistical analysis. The top 5 significantly enriched terms were reported for each cluster (FDR < 0.01).

##### Tissue-specific Epigenetic Cluster Profiling

The median log2 fold-change values used in Fig. 2*E* were calculated as follows: the median of the epigenetic signal of genes over all clusters in each tissue served as the expected value. The observed value was the median epigenetic signal in a given combination of cluster and tissue. Finally, a log2 observed/expected value was obtained showing the relative enrichment of the epigenetic signal between clusters for each tissue.

##### Calculating Epigenetic Similarity

Inverted Mahalanobis distance (1/Mahalanobis distance) was used to calculate the similarity between epigenetic profiles of genes. The “mahalanobis” R function was used with a user-specified covariance matrix.

##### Calculation of Gene Positional Clustering

Distances between all possible pairs of genes located on same chromosomes were calculated. For each gene, the mean distance to its 5 nearest neighbors was calculated. The list of genes was sorted by increasing mean distance to their 5 nearest neighbors. Finally, the genes at the top and bottom 5% of the list were labeled as most and least positionally clustered, respectively.

##### Calculation of Gene Expression Variability

Gene expression variability at the mRNA and protein levels was calculated as the coefficient of variation (CV; standard deviation divided by the mean) of log2-transformed TPM and SILAC ratios. To avoid dividing by zero (for unchanged genes with a log2 ratio of zero), a constant value of 10 was added to all mRNA and protein log2 ratios before calculating the variability.

##### Data Processing and Plotting

All data processing was performed in R (26) and the plots made using the ggplot2 package (27). The R scripts used to analyze data and generate most of the figures can be found on our GitHub (https://github.com/Rappsilber-Laboratory/tissue_mRNA_protein_scripts_MCP).

## RESULTS AND DISCUSSION

### 

#### 

##### Coexpression of Nearby Gene Pairs Is Buffered at the Protein Level in Mouse Tissues

**Fig. 1.**
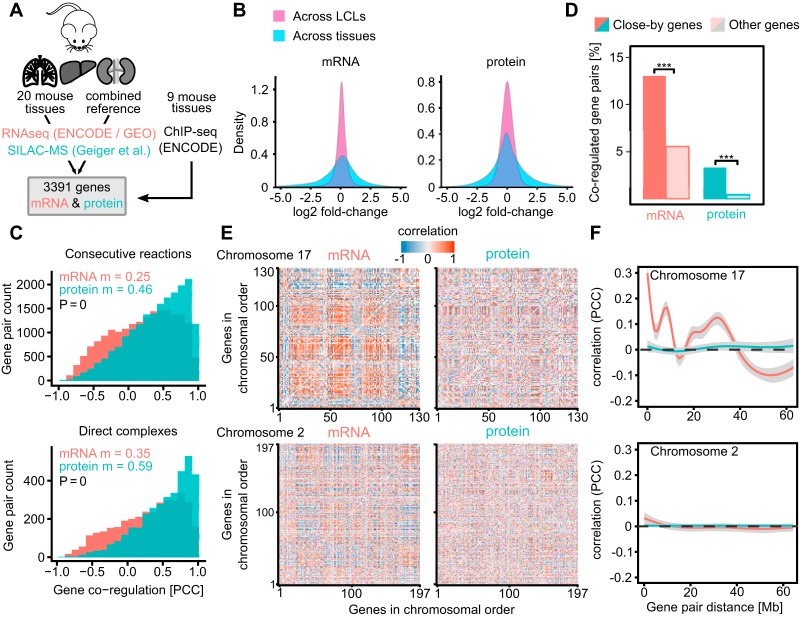
**Genomic distance between gene pairs affects their coexpression stronger on the mRNA than on the protein level.** *A*, We analyzed mRNA and protein expression changes between 20 different mouse tissues. Additionally we analyzed epigenetic profiles of genes by using ENCODE data for 9 different tissues. *B*, The global log2-fold changes in the mouse tissue data set are larger on both mRNA and protein levels compared with the LCL data set as used in (7). *C*, The coregulation of enzymes catalyzing consecutive metabolic reactions and protein complexes is significantly stronger on protein level compared with mRNA level (Mann-Whitney test *p* value < 0.0001 in both cases, m = median). *D*, The fraction of close-by genes (< 50 kilobases separation) coregulated on mRNA level is four times as large as on protein level which suggests that only about a quarter of the proximal mRNA coregulation is functional. Statistical significance was assessed using a Pearson's Chi-squared test (***p value < 0.0001). *E*, Chromosomal gene coregulation patterns are visible on mRNA level but disappear on protein level on chromosome 17. However, this effect seems not to be as strong for chromosome 2. *F*, The mRNA coregulation decreases with the linear gene separation albeit not monotonously, reflecting the observed chromosomal coregulation patches on chromosome 17. This effect is not observed on protein level. No long-range effects can be observed for chromosome 2. The gray area around the lines signifies 95% confidence intervals.

We assembled a mouse tissue expression data set comprising 3391 genes in 20 different tissues by combining proteomics and transcriptomics from different sources. Protein abundance data were derived from a quantitative proteomics data set based on metabolic isotope labeling of mice (10). Transcriptomics data were obtained from the ENCODE Consortium (11) and Gene Expression Omnibus (GEO) repository (12) (Fig. 1*A*). The tissue collection comprises few main broad functional categories such as the nervous system (cerebellum, brain cortex), digestive system (stomach, intestine, pancreas), immune system (thymus, spleen) and multifunctional organs such as the liver and kidney. To compare the gene expression between multiple tissues with enough statistical power, we used only genes expressed ubiquitously in all tissues as opposed to using tissue-specific genes. These so-called housekeeping genes account for about half of the genome in human (28) and presumably also in mouse. They are involved in basic cellular functions such as energy metabolism (including mitochondrial proteins), genome integrity maintenance, gene expression, protein trafficking, and cell structural functions.

To generate a coexpression matrix for all observed gene pairs on both mRNA and protein level, we calculated their Pearson correlation coefficients (PCCs) across the 20 tissues (exemplified in supplemental Fig. S1). Importantly, compared with a previous study on lymphoblastoid cell lines (LCLs) (7), the expression changes observed between tissues and consequently many different cell types were substantially larger (fold-change increased by a mean of ∼75% for both mRNA and proteins, Fig. 1*B*). We then assessed the quality and information content of the integrated data set by plotting the mRNA- and protein-level correlations for functionally related gene pairs. As expected, functional gene pairs have much higher correlation coefficients than randomly shuffled gene pairs (supplemental Fig. S2). This effect is more pronounced on protein than mRNA level (Fig. 1*C*). Subunits of the same complex correlated to a median of 0.59 at protein level and 0.35 at mRNA level. For comparison, in lymphoblastoid cell lines we observed 0.61 and 0.27, respectively. As one would expect, mRNA coexpression appears to be closer linked to function across tissues than closely related cell lines. Nevertheless, protein coexpression remains more indicative of shared function.

Next, we wondered about the impact of gene proximity on their correlated expression. We took gene pairs separated by less than 50 Kb between their transcription start sites (“close-by genes”) and looked at their mRNA correlation compared with gene pairs further apart (Fig. 1*D*). We observe 13% of close-by genes to have coregulated mRNAs. However, only a quarter of these (3.3%) are also coregulated on the protein level. This suggests that only a fraction of those coregulated mRNA pairs is functionally related. It is worth noting that even though our mRNA and protein data have similar numbers of data points per gene, the protein data is slightly sparser (15.5% and 6.7% missing values, respectively). Despite the numerical disadvantage of the protein data set, protein-level correlations are still more informative on the function than mRNA (Fig. 1*C*, supplemental Fig. S2). The data also differs in their measurement-based variation as they were acquired by different technologies. However, we are limiting our comparisons in most cases to within-mRNA and within-protein, avoiding direct mRNA-protein comparison.

As a second line of inquiry into the impact of gene proximity on their correlated expression, we grouped the gene pairs by chromosomes, arranged them in their genomic order and plotted their correlation values as a coregulation map (Fig. 1*E*). Patches of coregulated mRNAs are clearly visible on chromosome 17 that are not reflected on the protein level. The patches are seen along the diagonal, suggesting that neighboring genes tend to be cotranscribed. Patches are also found away from the diagonal. These patches likely reflect large-scale 3D architecture as we have shown in human (7). Fitting a generalized additive model (GAM) to the linear correlation data further highlights the observed coregulation patches which might be indicative of the chromosome folding (Fig. 1*F*, chromosome 17). The patches are not equally pronounced in all chromosomes, for example see chromosome 2 (Fig. 1*E*, 1*F*).

##### Gene Pairs with Sustained Coexpression Have Similar Post-transcriptional Regulation

**Fig. 2.**
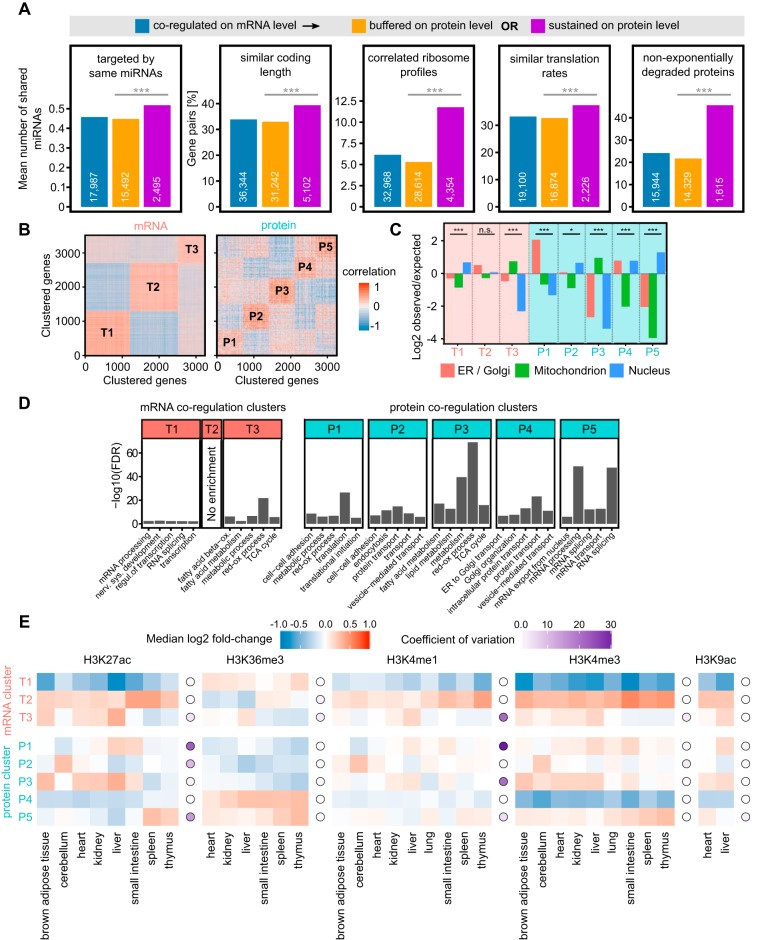
**mRNA and protein coregulation clusters are functionally distinct and display different epigenetic signatures.** *A*, Analysis of post-transcriptional regulation of gene pairs coexpressed on mRNA level. Gene pairs with sustained coexpression on the protein level share on average more miRNA targeting than pairs with buffered coexpression on the protein level (Mann Whitney *p* value < 0.0001). Gene pairs were considered to have similar CDS length if the ratio of the longer sequence to the shorter was < 1.5. Gene pairs were considered to have correlated ribosome profiles if their ribosome occupancy profiles had Pearson correlation coefficient > 0.5 (Holm adj. *p* value < 0.001). Gene pairs were considered to have similar translation rates if the absolute log2 ratio of their translation rates was lower or equal 1. For the non-exponentially degraded proteins (NEDs) bar chart, gene pairs containing at least one NED were counted. *B*, K-means clustering of the mRNA and protein coexpression data. Three distinct mRNA clusters and five distinct proteins clusters explained ∼50% of the variance in the respective data. *C*, mRNA coregulations clusters (T1–T3) have lower protein subcellular localization enrichments than protein coregulation clusters (P1–P5). The significance of enrichments/depletions in each cluster was tested using Pearson's Chi-squared test. ***p value < 0.0001, *p value < 0.05, n.s. = not significant. *D*, GO enrichment analysis of the genes in the mRNA and protein coregulation clusters. More GO terms are enriched in protein than in mRNA clusters. *E*, mRNA-based clusters T1 and T2 have uniform epigenetic signal distributions displaying little between-tissue variability as opposed to protein clusters which show large between-tissue and between-cluster variability. Epigenetic signal enrichment in tissue (squares), coefficient of variation for each histone mark (circle), color code as shown.

For many gene pairs, protein coexpression correlates with mRNA coexpression, while for other gene pairs mRNA and protein coexpression are not correlated. To identify possible mechanisms leading to buffered or sustained gene coexpression we conducted an analysis of post-transcriptional mechanisms using five published data sets (Fig. 2*A*). First, we looked at how many miRNAs are shared between gene pairs. miRNAs have been implicated in post-transcriptional gene expression control by binding to transcripts and regulating mRNA degradation and protein translation (29). Using miRNA-gene interaction data generated using the CLEAR-CLIP protocol (19), we found that gene pairs with sustained coexpression tend to share significantly more miRNAs than pairs with buffered coexpression (Mann Whitney *U* test *p* value = 0.002). We then looked at protein coding sequence (CDS) lengths which are a general indicator of the extent of post-transcriptional control (30). Gene pairs with sustained coexpression had significantly (Chi-squared Test *p* value < 0.0001) more similar CDS lengths than gene pairs with buffered coexpression patterns. Subsequently, we looked at levels of ribosome occupancy using ribosome profiling data from mouse liver (20) and protein translation rates determined using mass spectrometry (22). In both cases, gene pairs with sustained coexpression tend to have similar translation levels (Chi-squared Test *p* values < 0.0001 in both cases). Finally, we looked at protein degradation profiles by considering gene pairs having at least one nonexponentially degraded protein (NEDs) (23). We found that gene pairs with sustained coexpression are significantly enriched in NEDs (Chi-squared Test *p* value < 0.0001). Together, this suggests that various post-transcriptional mechanisms are involved in propagating functional gene coexpression to the protein level.

##### Protein Coregulation Clusters Are More Functional Than mRNA Coregulation Clusters

To group genes with similar coexpression patterns we used k-means clustering (Fig. 2*B*). This expands our analysis of coregulation from gene pairs to gene groups. This revealed specific coregulation patterns in which each cluster tends to be coregulated or antiregulated with other clusters (supplemental Fig. S3). Of the three transcript-based gene clusters, cluster T1 and T2 are anticorrelated. A similar anticorrelation was observed in human, which could be traced there to chromosome subcompartments A1 and A2 (7). Briefly, compartments are regions of the genome defined by 3D analysis of chromosome structure (31). Compartment A is characterized by active gene expression whereas compartment B mostly by suppressed gene expression. It was later discovered that both A and B compartments are divided further into subcompartments A1, A2 and B1 to B4, each with distinct epigenetic marks and spatial interaction patterns (32).

In the absence of equivalent high-resolution HiC data for mouse tissues we tested for epigenetic similarity within these clusters as epigenetic signatures closely link to chromatin subcompartments (32). Indeed, the epigenetic signatures of T1 and T2 clusters resemble those found in chromatin subcompartments A1 and A2 (see next paragraph). Notably, neither in mouse nor in human do the transcript-based gene clusters inform on protein coexpression. The marked exception is given by cluster T3 which displays coexpression behavior also at the protein level. Looking at the function of genes present in each of the clusters by performing subcellular localization (Fig. 2*C*) and Gene Ontology (33) term enrichment (Fig. 2*D*) reveals that cluster T3 is enriched for mitochondrial functions. This indicates large differences in the energetic needs of different tissues, which may require gene regulation at both the transcriptional and protein level. The five protein-based gene clusters correlate with each other to various degrees, with the anti-correlations of P2 *versus* P4 and P3 *versus* P5 being most pronounced. These likely reflect commitments of cell types to different large cellular processes (Fig. 2*D*). Interestingly, we observed a large overlap between the clusters T3 and P3. They had 734 and 686 members, respectively, and around half of the members were shared between them (365 genes). Similarly, to cluster T3, the protein cluster P3 was enriched in mitochondrial functions (Fig. 2*C*, 2*D*). This suggests that the coordination of mitochondrial protein coexpression could be tightly controlled already on the mRNA level.

Except for P3, the protein-based gene clusters are not reflected in transcript coexpression (supplemental Fig. S3). In summary, one of the three transcript-based gene clusters show some functional enrichment. However, all five protein coregulation clusters show well-defined subcellular localization patterns and functional GO term enrichments. As observed in other systems, protein coexpression links closer to function than transcript coexpression (7, 8).

We added a regulatory dimension to the expression data set by leveraging the ENCODE ChIP-seq data resources for nine different mouse tissues. This allowed us to estimate epigenetic variability of the gene pairs in the data. We calculated ChIP-seq signal enrichment for gene bodies belonging to the mRNA and protein coregulation clusters (Fig. 2*E*). Transcript clusters T1 and T2, which cover about 80% of the genes, maintain their epigenetic profile across all tissues with T2 being more enriched in activating marks compared with T1. While these two groups are defined through their chromatin state, they do not experience tissue specific regulation through epigenetic processes. This might be linked to chromatin subcompartments. Indeed, the epigenetic patterns of mouse clusters T1 and T2 closely resemble human chromatin subcompartments A2 and A1, respectively (7). This suggests a similar chromatin subcompartmentalization in mouse as is found in human. In contrast, transcript cluster T3 and most protein clusters display epigenetic variation across tissues indicating the action of an epigenetic program which is in line with epigenetic processes being involved in cell differentiation (34). It may initially surprise that protein clusters have epigenetic tissue-specific changes while transcript clusters T1 and T2 lack these (for example see H3K27ac or H3K4me1). This is consistent with subcompartments dominating the epigenetic signature that is associated with mRNA coexpression. It is worth keeping in mind that we analyze housekeeping genes, for which one would expect adjustments in expression rather than on/off changes and consequently only weak epigenetic influences. Interestingly, a strong between-cluster difference can be seen for the H3K36me3 mark which displays almost no variability between tissues for protein clusters. The H3K36me3 mark has been shown to be implicated in gene expression noise control through a mechanism of transcriptional burst frequency modulation (35) and to be enriched among noise-sensitive, highly expressed genes (36, 37). In full agreement with this, the mRNA cluster enriched in the H3K36me3 mark (T1) has significantly lower expression variability compared with other clusters (supplemental Fig. S4*A*). Curiously, we also observed a strong expression variability difference for protein clusters P4 and P5 which are enriched for H3K36me3 compared with other three protein clusters. However, it is not clear if the differences in H3K36me3 signal in mRNA and protein clusters are a cause of different expression variability or an effect of differences in the ongoing transcription.

##### Gene Clustering Reduces mRNA Expression Variability in Mouse Tissues

**Fig. 3.**
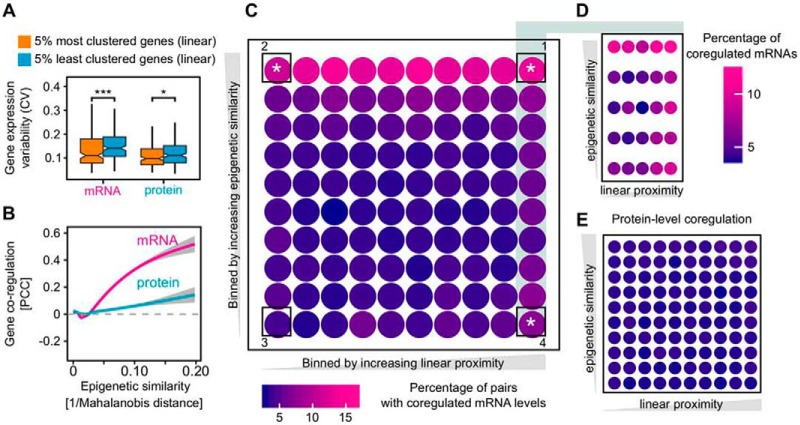
**The impact of gene proximity and epigenetic similarity on mRNA- and protein-level coregulation.** *A*, Positional gene clustering reduces the expression variability on mRNA level. We calculated the expression variability (coefficient of variation, CV) of the 5% most and 5% least positionally clustered genes on the genome (*i.e.* considering their sequence proximity). The difference is significant (using Mann-Whitney test) on both mRNA level (***p value = 0.00029) and protein level (*p value = 0.019). When using 10 and 1% most and least clustered genes, we obtain the same statistical results as with 5% (data not shown). Boxplot drawn in the style of Tukey, *i.e.* box limits indicate the first and third quartiles, central lines the median, whiskers extend 1.5 times the interquartile range from the box limits. Notches indicate the 95% confidence interval for comparing medians. *B*, Gene coregulation increases with epigenetic similarity at the mRNA level, whereas it remains largely independent from epigenetic similarity at the protein level. *C*, Epigenetic similarity is the major driver of the mRNA coregulation. Gene pairs were considered coregulated if their mRNA level correlation was > 0.5 and the BH-adjusted *p* values < 0.05. The bins were created by dividing gene pair distances and epigenetic similarity (1/Mahalanobis distance) into 10 roughly equal sets. This yielded 100 unique bin combinations. The color signifies the percentage of coregulated mRNA in each bin. The mean gene pair distance in the left-most column is 115 Mb and 2 Mb in the right-most column. White stars (*) mark corner sectors which have significantly higher mRNA coexpression compared with an equal-sized random background sample as judged by Kolmogorov-Smirnov test. The procedure was repeated 1000 times. The mean *p* values for sectors 1, 2, 3 and 4 were 0, 10^−13^, 0.039 and 6*10^−9^, respectively. *p* value of 0 is reported by the KS test for extremely low values. *D*, Effects in linear distance are confined to very close proximity. The 10 bins constituting the right-most column in Fig. 3*C* were extracted and magnified. The mean gene pair distance for the left-most column is 4 Mb and 240 Kb for the right-most column. *E*, Protein-level coregulation of housekeeping genes is not generally affected by epigenetic similarity or linear distance.

We determined the gene expression variability (coefficient of variation, CV) of the most and least densely clustered genes, considering sequence proximity (Fig. 3*A*). Transcript expression variability is reduced significantly for genes clustered in the genome sequence while the effect is less pronounced for protein expression variability. Importantly, although gene expression variability generally covariates with expression level, no difference in expression levels was observed here for the top and lowest 5% positionally clustered genes (53,000 and 56,000 mean TPM, respectively). As observed previously for yeast (38) and human (7) gene clustering may safeguard against accidental silencing and the resulting expression noise. However, gene expression variability is not exactly the same as bona fide gene expression noise. It is interesting therefore that our observations using global between-tissue variability of expression reflect the observations based on expression noise in its classical sense in other systems. As a further link of expression variability between tissues to noise, we noted a strong dependence of both mRNA and protein expression variability on H3K36me3 signal in gene bodies. Genes lacking H3K36me3 signal are the most variably expressed between the tissues whereas the opposite is true for genes with strong H3K36me3 signal (supplemental Fig. S4*B*). This resembles the role of this mark in expression noise control (36, 37).

##### Epigenetic Similarity Is the Main Driver of Nonfunctional mRNA Coexpression

Coexpression of close-by, unrelated genes can be driven by at least two distinct mechanisms. First, stochastic fluctuations between the on and off state of a chromatin domain can affect multiple genes simultaneously and lead to their coexpression (4, 39). In addition, coexpression can reflect a transcriptional “ripple effect,” where the activation of one gene leads to the upregulation of other genes in its immediate neighborhood (5). We investigated which of these factors drives non-functional mRNA coexpression across mouse tissues. To estimate which genes may be affected by the same chromatin fluctuations, we first determined the epigenetic profile of each gene, based on 7 histone marks in 9 different tissues reported by ENCODE. We then calculated the epigenetic similarity between all gene pairs using the Mahalanobis distance, which considers that some histone marks are codependent (exemplified in supplemental Fig. S5). As one might expect, we observed that correlation of mRNA abundances increases dramatically with increasing epigenetic similarity of their respective genes. Interestingly, the effect is largely buffered on the protein level (Fig. 3*B*). This suggests that many mRNA pairs are coactivated as a side-effect of their genes being in the same genomic neighborhood which in turn confers a specific epigenetic profile. To place the epigenetic similarity and coregulation into gene position context, we plotted the coregulation values as a function of both epigenetic similarity and a linear genomic separation of the gene pairs (Fig. 3*C*). Strikingly, epigenetic similarity drives mRNA coexpression irrespective of whether genes are far apart (Fig. 3*C*, sector 2) or close-by in the genome (sector 1). For the gene pairs that are on average within 2 Mb to each other, those that have very different epigenetic profiles are much less likely to be coexpressed than those with similar chromatin features (Fig. 3*C*, sector 4 *versus* 1). This is most likely an effect of global fluctuations of chromatin factors shown previously in yeast (40). Gene proximity only starts to be a driving factor for genes less than 240 Kb apart (Fig. 3*D*, right-most column) which agrees with previous observations of a local transcriptional ripple effect (5). Notably, most of this mRNA coexpression is non-functional, because the same group of genes show, on average, no coexpression at the protein level (Fig. 3*E*).

## CONCLUSIONS

In an LCL cell line panel and in cancer samples, at homeostatic conditions much of mRNA coexpression is non-functional, *i.e.* does not affect protein coexpression and instead can be traced back to genome organization. We wondered how much coexpression of mRNA and proteins would be linked when comparing very different cellular states given by multiple fully differentiated tissues. mRNA coexpression is indeed more closely linked to function in mouse tissues than in homeostatic conditions, although protein coexpression is significantly more indicative of function. The epigenetic profiling of coexpression clusters revealed that mRNA coexpression is affected by two distinct epigenetic states, most likely reflecting the different genomic subcompartments in which they reside. As observed in homeostatic conditions, this broad positioning effect on mRNA coexpression is then buffered on the protein level. However, in mouse tissues the non-functional mRNA coexpression is linked more closely to epigenetic states than to linear gene proximity. Epigenetic differences between the tissues dwarf the linear proximity effect on coexpression. Notably, we chose to use housekeeping genes only as they conferred enough data points to be usable in this correlation-based study. It is not clear to what extent do the observations on housekeeping genes generalize to the rest of the genome. Taken together, our observations lend support to the notion of monitoring protein coexpression for functional genomics. However, to fully understand the impact of epigenetics on mRNA and protein coexpression and the underlying mechanisms, more in-depth experimental studies are needed.

## Supplementary Material

supplemental Table S1
