# Supplementary material for: Epigenetic Variability Confounds Transcriptome but Not Proteome Profiling for Coexpression-based Gene Function Prediction
Source: Mol Cell Proteomics. 2018 Jul 24;17(11):2082–90. doi: 10.1074/mcp.RA118.000935 (PMC6210221; doi:10.1074/mcp.RA118.000935)
Supplement: supplemental Table S1 [file RA118.000935_index.html]

Supplement to Epigenetic Variability Confounds Transcriptome but not Proteome Profiling for Coexpression-based Gene Function Prediction | Molecular & Cellular Proteomics

## Supplemental Data

- Table S1 - Links to RNAseq data (ENCODE and GEO)
- Supplemental File 1 - Protein and mRNA expression matrix used in the study
- Supplemental File 2 - Processed ChIP-seq signal matrix used in the study
- Supplemental\_Figure\_1 - Supplemental\_Figure\_1
- Supplemental\_Figure\_2 - Supplemental\_Figure\_2
- Supplemental\_Figure\_3 - Supplemental\_Figure\_3
- Supplemental\_Figure\_4 - Supplemental\_Figure\_4
- Supplemental\_Figure\_5 - Supplemental\_Figure\_5
