# Supplementary material for: Epigenetic Variability Confounds Transcriptome but Not Proteome Profiling for Coexpression-based Gene Function Prediction
Source: Mol Cell Proteomics. 2018 Jul 24;17(11):2082–90. doi: 10.1074/mcp.RA118.000935 (PMC6210221; doi:10.1074/mcp.RA118.000935)
Supplement: supplemental Table S1 [file 138987_1_supp_165910_pblqbg.pdf]

Supplemental Table S1

List of RNAseq experiments and sources of downloaded data.

Tissue type and details:	Source:
**Adrenal gland**, *mus musculus*, mixed sex littermates, adult, 8 weeks	ENCODE<br>https://www.encodeproject.org/experiments/ENCSR000BYX<br><br>Ref: http://www.ncbi.nlm.nih.gov/pubmed/25409824
**Brain cortex**, *mus musculus,* males, adult, 8 weeks	ENCODE<br>https://www.encodeproject.org/experiments/ENCSR000CLJ<br><br>Ref: http://www.ncbi.nlm.nih.gov/pubmed/25409824
**Brown adipose tissue**, *mus musculus,* males, adult, 24 weeks	ENCODE<br>https://www.encodeproject.org/experiments/ENCSR000CHN<br><br>Ref: http://www.ncbi.nlm.nih.gov/pubmed/25409824
**Cerebellum**, *mus musculus,* mixed sex littermates, adult, 8 weeks	ENCODE<br>https://www.encodeproject.org/experiments/ENCSR000BZM<br><br>Ref: http://www.ncbi.nlm.nih.gov/pubmed/25409824
**Duodenum**, *mus musculus,* mixed sex littermates, adult, 8 weeks	ENCODE<br>https://www.encodeproject.org/experiments/ENCSR000BYY<br><br>Ref: http://www.ncbi.nlm.nih.gov/pubmed/25409824
**Embryonic tissue**, *mus musculus,* males, embryonic, 11.5 days	ENCODE<br>https://www.encodeproject.org/experiments/ENCSR000CLL<br><br>Ref: http://www.ncbi.nlm.nih.gov/pubmed/25409824
**Eye**, *mus musculus,* females, adult, 4 weeks<br><br>Note: wild-type control data used	GEO<br>http://www.ncbi.nlm.nih.gov/geo/query/acc.cgi?acc=GSM737548<br><br>Ref: http://www.ncbi.nlm.nih.gov/pubmed/21659555
**Heart**, *mus musculus,* mixed sex littermates, adult, 8 weeks	ENCODE<br>https://www.encodeproject.org/experiments/ENCSR000BYQ<br><br>Ref: http://www.ncbi.nlm.nih.gov/pubmed/25409824

**Kidney**, *mus musculus,* mixed sex littermates, adult, 8 weeks	ENCODE<br>https://www.encodeproject.org/experiments/ENCSR000BYR<br><br>Ref: http://www.ncbi.nlm.nih.gov/pubmed/25409824
**Large intestine**, *mus musculus,* mixed sex littermates, adult, 8 weeks	ENCODE<br>https://www.encodeproject.org/experiments/ENCSR000BZA<br><br>Ref: http://www.ncbi.nlm.nih.gov/pubmed/25409824
**Liver**, *mus musculus,* mixed sex littermates, adult, 8 weeks	ENCODE<br>https://www.encodeproject.org/experiments/ENCSR000BYS<br><br>Ref: http://www.ncbi.nlm.nih.gov/pubmed/25409824
**Lung**, *mus musculus,* mixed sex littermates, adult, 8 weeks	ENCODE<br>https://www.encodeproject.org/experiments/ENCSR000BYT<br><br>Ref: http://www.ncbi.nlm.nih.gov/pubmed/25409824
**Ovary**, *mus musculus*, females, adult, 8 weeks	ENCODE<br>https://www.encodeproject.org/experiments/ENCSR000BZC<br><br>Ref: http://www.ncbi.nlm.nih.gov/pubmed/25409824
**Pancreas**, *mus musculus,* females, adult, 12 weeks	GEO<br>http://www.ncbi.nlm.nih.gov/geo/query/acc.cgi?acc=GSE21860<br><br>Ref: http://www.ncbi.nlm.nih.gov/pubmed/20581837
**Skeletal muscle**, *mus musculus*, male, adult, 8 weeks	ENCODE<br>https://www.encodeproject.org/experiments/ENCSR000CME<br><br>Ref: http://www.ncbi.nlm.nih.gov/pubmed/25409824
**Spleen**, *mus musculus,* male, adult, 8 weeks	ENCODE<br>https://www.encodeproject.org/experiments/ENCSR000CGW<br><br>Ref: http://www.ncbi.nlm.nih.gov/pubmed/25409824
**Stomach**, *mus musculus,* mixed sex littermates, adult, 8 weeks	ENCODE<br>https://www.encodeproject.org/experiments/ENCSR000BZE<br><br>Ref: http://www.ncbi.nlm.nih.gov/pubmed/25409824

**Subcutaneous adipose tissue**, *mus musculus,* mixed sex littermates, adult, 8 weeks	ENCODE<br>https://www.encodeproject.org/experiments/ENCSR000BZF<br><br>Ref: http://www.ncbi.nlm.nih.gov/pubmed/25409824
**Thymus**, *mus musculus,* mixed sex littermates, adult, 8 weeks	ENCODE<br>https://www.encodeproject.org/experiments/ENCSR000BYV<br><br>Ref: http://www.ncbi.nlm.nih.gov/pubmed/25409824
**Uterus**, *mus musculus,* females, immature, 25 days<br><br>Note: wild-type control samples used	GEO<br>http://www.ncbi.nlm.nih.gov/geo/query/acc.cgi?acc=GSE47019<br><br>Ref: http://www.ncbi.nlm.nih.gov/pubmed/24503642
