# Supplementary material for: Epigenetic Variability Confounds Transcriptome but Not Proteome Profiling for Coexpression-based Gene Function Prediction
Source: Mol Cell Proteomics. 2018 Jul 24;17(11):2082–90. doi: 10.1074/mcp.RA118.000935 (PMC6210221; doi:10.1074/mcp.RA118.000935)
Supplement: supplemental Table S1 [file 138987_1_supp_165906_pblqbg.pdf]

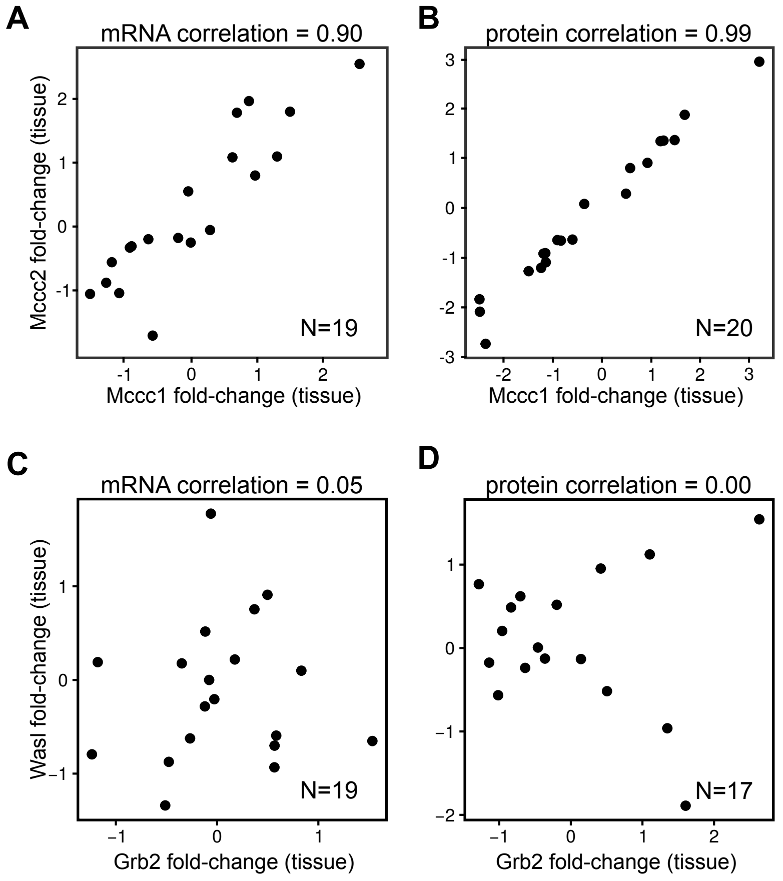

Supplemental Figure S1. High and low correlated gene pairs

Examples of highly correlated gene pairs on mRNA level (A) and protein level (B) and non-correlated gene pairs on mRNA level (C) and protein level (D). Each data point is a fold-change of expression value of a gene in one tissue relative to the mean of all tissues.
N = data points used for correlation estimation.
