# Supplementary material for: Epigenetic Variability Confounds Transcriptome but Not Proteome Profiling for Coexpression-based Gene Function Prediction
Source: Mol Cell Proteomics. 2018 Jul 24;17(11):2082–90. doi: 10.1074/mcp.RA118.000935 (PMC6210221; doi:10.1074/mcp.RA118.000935)
Supplement: supplemental Table S1 [file 138987_1_supp_165904_pblqbg.pdf]

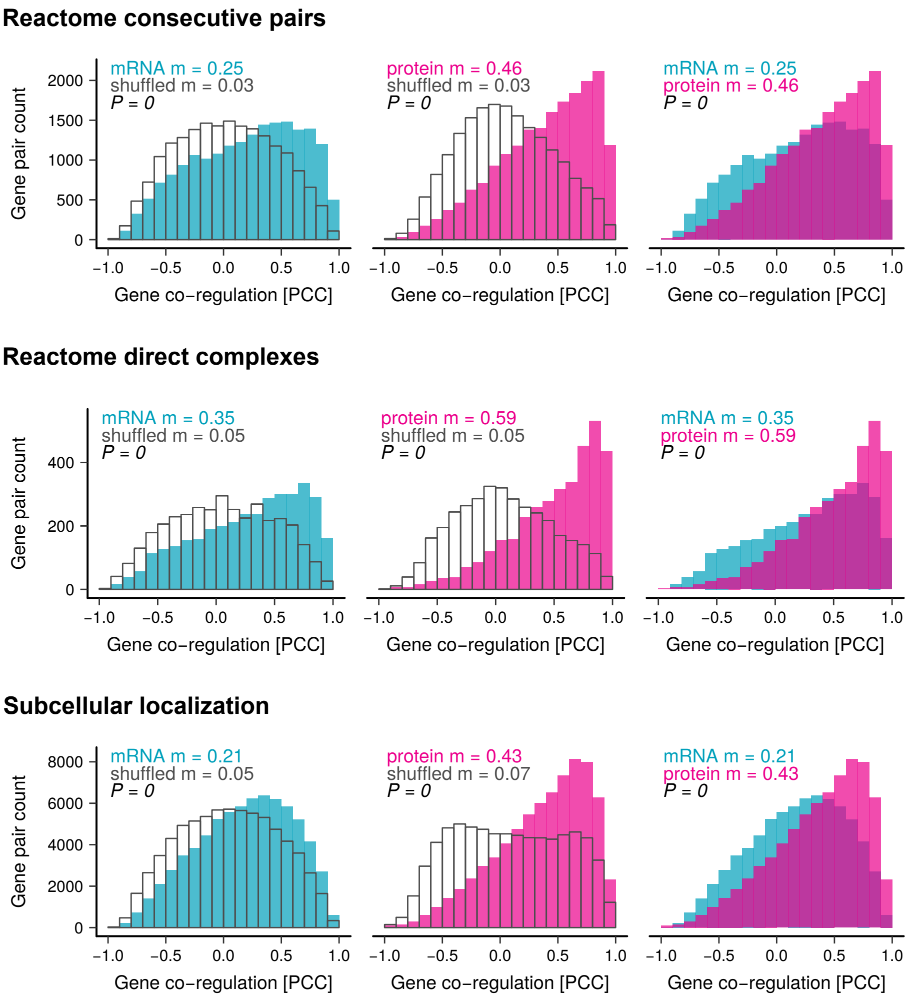

Supplemental Figure S2. mRNA and protein correlations of functionally-related groups

Correlation distributions for functional gene pairs based on Reactome (genes coding proteins involved in consecutive steps in metabolic pathways and direct complexes) and Uniprot (genes coding proteins with same subcellular localisation). In each group protein-level correlations were significantly stronger than on mRNA level ($P < 0.0001$, Kolmogorov–Smirnov test). Both mRNA and protein correlations for functional pairs were significantly higher than for random pairs (shuffled).
