# Supplementary material for: Epigenetic Variability Confounds Transcriptome but Not Proteome Profiling for Coexpression-based Gene Function Prediction
Source: Mol Cell Proteomics. 2018 Jul 24;17(11):2082–90. doi: 10.1074/mcp.RA118.000935 (PMC6210221; doi:10.1074/mcp.RA118.000935)
Supplement: supplemental Table S1 [file 138987_1_supp_165905_pblqbg.pdf]

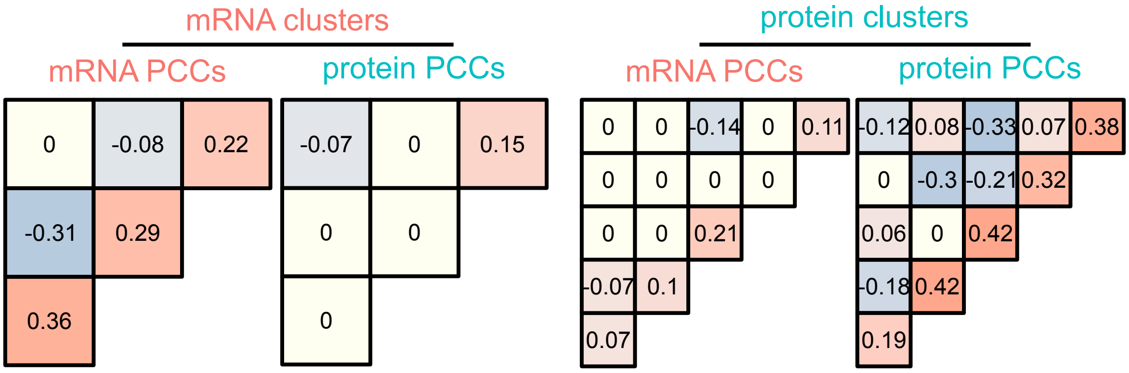

Supplemental Figure S3 Median Pearson correlation coefficients (PCCs) of co-regulation clusters.

Using median protein PCCs grouped by mRNA clusters (and vice versa) shows that there is a limited relation between the mRNA and protein clusters. The absolute PCC values ≤ 0.05 were rounded down to 0 for visual clarity.
