# Supplementary material for: Epigenetic Variability Confounds Transcriptome but Not Proteome Profiling for Coexpression-based Gene Function Prediction
Source: Mol Cell Proteomics. 2018 Jul 24;17(11):2082–90. doi: 10.1074/mcp.RA118.000935 (PMC6210221; doi:10.1074/mcp.RA118.000935)
Supplement: supplemental Table S1 [file 138987_1_supp_165907_pblqbg.pdf]

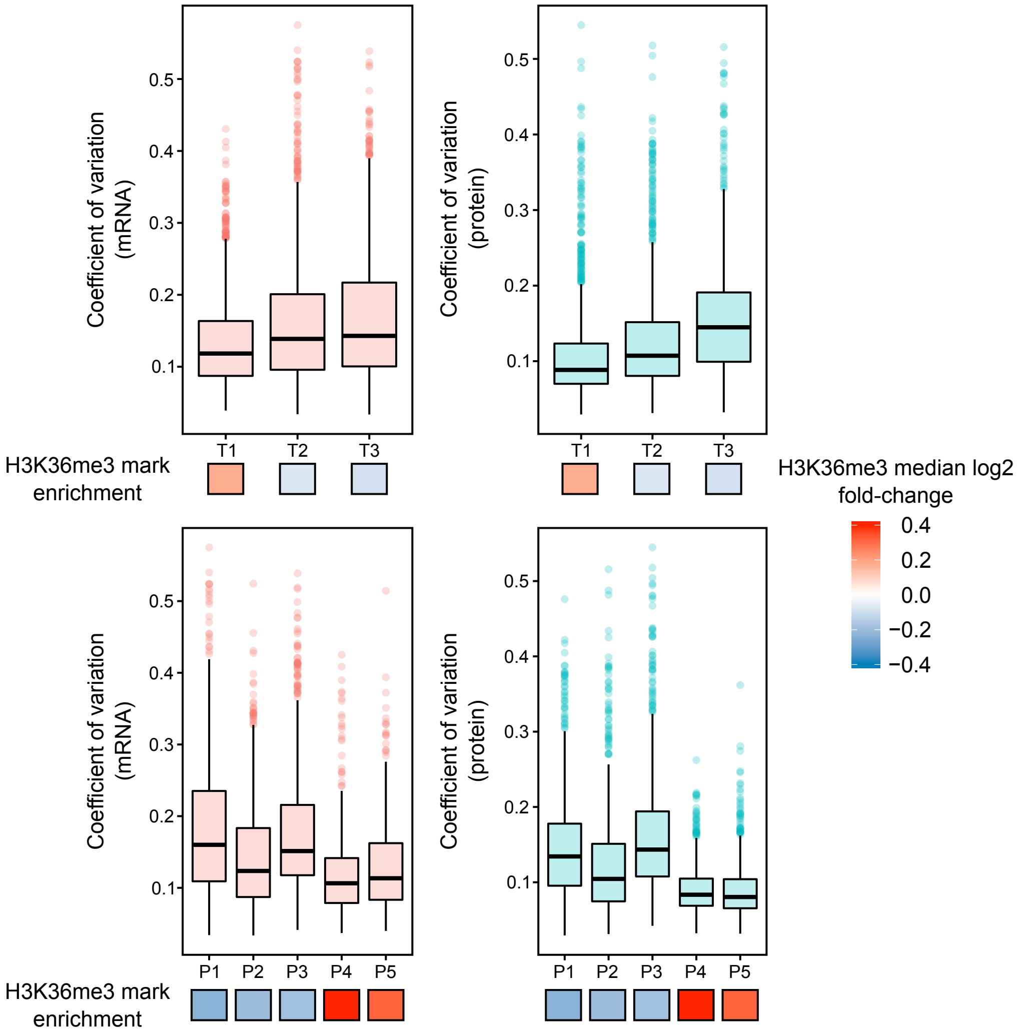

Supplemental Figure S4 A H3K36me3 signal is enriched in clusters with lowest expression variability (clusters T1, P4 and P5). The gene expression variability is significantly lower for T1 cluster compared to T2 (Kolmogorov-Smirnov test P-value < 0.0001).

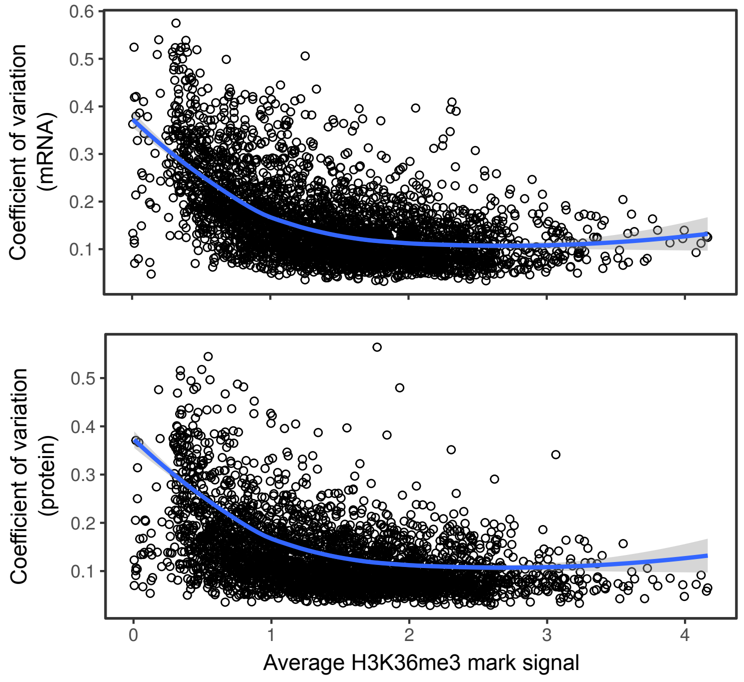

Supplemental Figure S4 B Genes with the highest expression variability on both mRNA and protein level have the lowest H3K36me3 signal.
