# Supplementary material for: Epigenetic Variability Confounds Transcriptome but Not Proteome Profiling for Coexpression-based Gene Function Prediction
Source: Mol Cell Proteomics. 2018 Jul 24;17(11):2082–90. doi: 10.1074/mcp.RA118.000935 (PMC6210221; doi:10.1074/mcp.RA118.000935)
Supplement: supplemental Table S1 [file 138987_1_supp_165903_pblqbf.pdf]

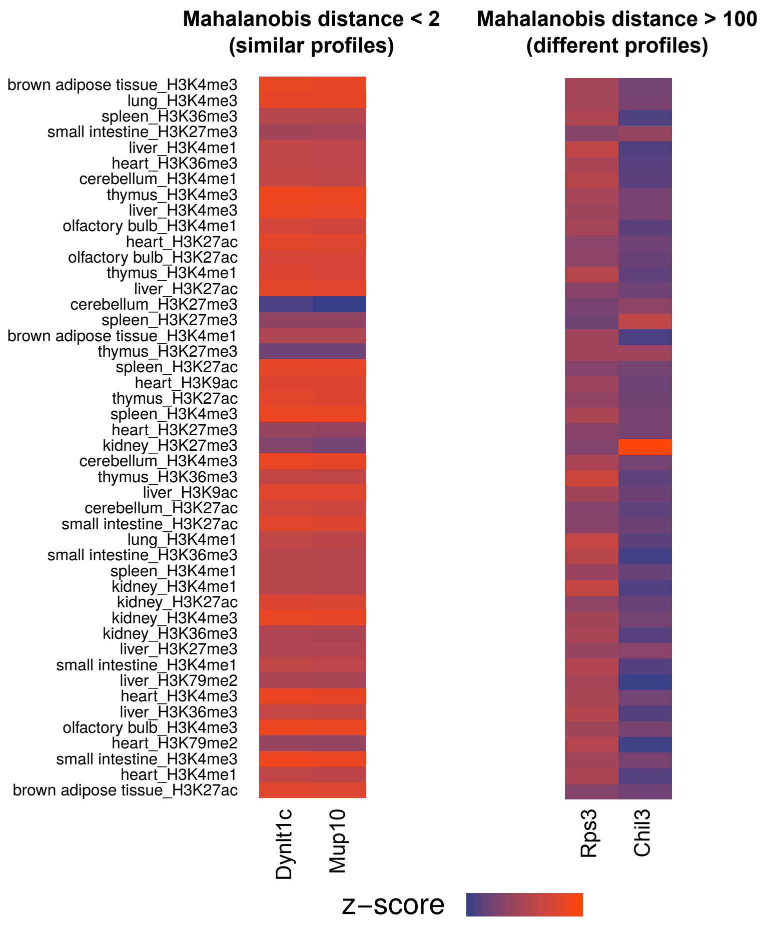

Supplemental Figure S5. Examples of epigenetically similar and dissimilar gene pairs.

Mahalanobis distance was used to assess similarities of tissue-specific epigenetic signals for gene pairs. The robustness of this analysis is conferred by many data points for each gene.
